# Penpulimab for Relapsed or Refractory Classical Hodgkin Lymphoma: A Multicenter, Single-Arm, Pivotal Phase I/II Trial (AK105-201)

**DOI:** 10.3389/fonc.2022.925236

**Published:** 2022-07-07

**Authors:** Yuqin Song, Keshu Zhou, Chuan Jin, Zhengzi Qian, Ming Hou, Lei Fan, Fei Li, Kaiyang Ding, Hui Zhou, Xiaoling Li, Bing Chen, Xiuhua Sun, Xianmin Song, Ming Jiang, Qingyuan Zhang, Lihong Liu, Guohua Yu, Yu Hu, Zheng Zhao, Ligen Liu, Hongwei Xue, Jun Luo, Bai He, Xiaoping Jin, Min Zhao, Baiyong Li, Yu Xia, Jun Zhu

**Affiliations:** ^1^Key Laboratory of Carcinogenesis and Translational Research (Ministry of Education), Department of Lymphoma, Peking University Cancer Hospital & Institute, Beijing, China; ^2^Department of Hematology, The Affiliated Cancer Hospital of Zhengzhou University and Henan Cancer Hospital, Zhengzhou, China; ^3^Department of Oncology, Cancer Hospital Affiliated to Guangzhou Medical University, Guangzhou, China; ^4^Department of Lymphoma, Tianjin Medical University Cancer Institute and Hospital, National Clinical Research Center of Cancer, Key Laboratory of Cancer Prevention and Therapy, the Sino-US Center for Lymphoma and Leukemia Research, Tianjin, China; ^5^Department of Hematology, Qilu Hospital, Shandong University, Jinan, China; Shandong Provincial Key Laboratory of Immunohematology, Qilu Hospital, Shandong University, Jinan, China; ^6^Department of Hematology, The First Affiliated Hospital with Nanjing Medical University, Jiangsu Province Hospital, Collaborative Innovation Center for Cancer Personalized Medicine, Nanjing, China; ^7^Department of Hematology, The First Affiliated Hospital of Nanchang University, Nanchang, China; ^8^Department of Hematology, The First Affiliated Hospital of USTC, Division of Life Sciences and Medicine, University of Science and Technology of China, Hefei, China; ^9^Lymphoma & Hematology Department, Tumor Hospital of Xiangya School of Medicine of Central South University, Changsha, China; ^10^Department of Medical Oncology, Liaoning Cancer Hospital and Institute, Shenyang, China; ^11^Department of Hematology, Nanjing Drum Tower Hospital, Clinical College of Nanjing Medical University, Nanjing, China; ^12^Department of Medical Oncology, Second Affiliated Hospital of Dalian Medical University, Dalian, China; ^13^Department of Hematology, Shanghai First People’s Hospital, Shanghai Jiaotong University, Shanghai, China; ^14^Department of Medical Oncology, Cancer Center, West China Hospital, Sichuan University, Chengdu, China; ^15^Department of Medical Oncology, Heilongjiang Provincial Hospital, Harbin, China; ^16^Department of Hematology, The Fourth Hospital of Hebei Medical University, Shijiazhuang, China; ^17^Clinical Oncology Department, Weifang People’s Hospital, Weifang, China; ^18^Institute of Hematology, Union Hospital, Tongji Medical College, Huazhong University of Science and Technology, Wuhan, China; ^19^Third Department of Medical Oncology, Shaanxi Provincial Cancer Hospital, Xi’an, China; ^20^Department of Hematology, Shanghai Tongren Hospital, Shanghai, China; ^21^Department of Hematology, The Affiliated Hospital of Qingdao University, Qingdao, China; ^22^Department of Hematology, The First Affiliated Hospital of Guangxi Medical University, Nanning, China; ^23^Department of Hematology, The Third Affiliated Hospital of Suzhou University, The First People’s Hospital of Changzhou, Changzhou, China; ^24^Akeso Biopharma Co., Ltd., Zhongshan, China

**Keywords:** IgG1 anti-PD-1 antibody, penpulimab, classical Hodgkin lymphoma, efficacy, safety

## Abstract

**Background:**

Nearly all anti-PD-1 antibodies are of the IgG4 isotype, and thus possess residual FcR effector functions. Such anti-PD-1 antibodies are also associated with immune tolerance and escape due to instability of the CH3 domain and Fc-Fc interaction. In this trial, we examined the efficacy and safety of penpulimab, a novel IgG1 anti-PD-1 antibody that does not bind to the Fc receptor, in patients with refractory or relapsed classical Hodgkin lymphoma (R/R cHL).

**Methods:**

Adult patients (≥18 years of age) with R/R cHL received 200 mg penpulimab once biweekly until disease progression or unacceptable toxicities for a maximum of 24 months. The primary endpoint was objective response rate (ORR) based on the Independent Radiology Review Committee per Lugano 2014 criteria. Secondary endpoints included progression-free survival (PFS), overall survival (OS), treatment-related adverse events (TRAEs) and immune-related adverse events (irAEs).

**Results:**

A total of 94 patients were enrolled. The median follow-up was 15.8 months. The ORR was 89.4% (95% CI 80.8%, 95.0%) in the full analysis set (85 patients). Forty (47.1%) patients achieved complete remission, 36 (42.4%) patients achieved partial remission. The 12-month PFS rate was 72.1% (95% CI 60.5%, 80.8%) and the 18-month OS rate was 100%. Totally 97.9% (92/94) of patients experienced at least one TRAE. The rate of grade 3 and above TRAEs was 26.6% (25/94). In addition, 51 (54.3%) patients experienced an irAE, and 4 (4.3%) patients developed grade 3 or above irAEs. No irAE-related death occurred.

**Conclusions:**

Penpulimab was effective and safe in patients with R/R cHL.

## Introduction

Classical Hodgkin lymphoma (cHL) is a malignant B cell lymphoma and occurs most commonly between 20 and 40 years of age (1). Despite a high clinical cure rate with first-line chemotherapy, approximately 5%-10% of the patients are refractory to initial chemotherapy. In addition, 10%-30% of patients who achieve complete remission (CR) with first-line chemotherapy eventually relapse (2). Though the antibody-drug conjugate brentuximab vedotin has achieved an objective response rate (ORR) of 75% in R/R cHL patients who have failed autologous stem cell transplant (ASCT), anti-PD-1 antibody pembrolizumab yielded significantly longer median progression-free survival (PFS) *versus* brentuximab vedotin in the KEYNOTE-204 trial [13·2 *vs.* 8·3 months, hazard ratio 0·65 (95% CI 0·48-0·88), *P*=0·0027] in R/R cHL patients who had relapsed post-ASCT or were ineligible for ASCT, indicating that PD-1 monoclonal antibodies are more efficacious than brentuximab vedotin in these patients (3).

Though nivolumab and pembrolizumab have demonstrated antitumor activities in R/R cHL patients (4–7), no sufficient data is available on the two drugs in Chinese cHL patients, and neither has been approved to treat cHL patients in China. Several PD-1 monoclonal antibodies, including tislelizumab, sintilimab, and camrelizumab, have received conditional approval in China for R/R cHL patients based on the results of single arm phase II trials (4–13). All these anti-PD-1 antibodies are IgG4 in nature whose fragment crystallizable (Fc) fragment can react to the Fc fragments of cancer-specific IgG1 bound to cancer antigens, interfering with IgG1-mediated effector functions and resulting in immune escape (14). Moreover, anti-PD-1 IgG4 antibody could engage FcγRI^+^ macrophages, induce antibody-dependent cell-mediated phagocytosis (ADCP), damage PD-1^+^ T cells and stimulate the release of IL-6, TNF-α, and other inflammatory cytokines, which may play a role in the development of immune-related adverse events (irAEs) (15–18).

Penpulimab, also known as AK105, is a human IgG1 anti-PD-1 antibody. A unique feature of penpulimab is a Fc mutation that eliminates Fc receptor and complement-mediated effector function, thus avoiding antibody-dependent cell-mediated cytotoxicity (ADCC), ADCP, and complement-dependent cytotoxicity (CDC), which are induced by binding of Fc to Fc receptor FcγRIIIa, FcγRIa, and C1q, respectively (19). In addition, IgG1 is structurally stable and thus minimizes immune escape (20, 21). A phase 1a dose-escalation trial of 16 patients with solid tumors found no dose-limiting toxicities (DLTs) of penpulimab up to the maximally allowed dose of 10 mg/kg and treatment-related adverse events (TRAEs) in 2 (12.5%) cases. Four patients achieved partial response (PR) and 4 patients achieved stable disease (SD), thereby indicating a favorable risk-benefit ratio. Given these promising results, we conducted this multicenter, single-arm, pivotal phase I/II trial to evaluate the efficacy and safety of penpulimab in Chinese patients with R/R cHL.

## Materials and Methods

### Patients

Adult patients (≥18 years of age) with pathologically proven R/R cHL were eligible. We included patients who had received combination chemotherapy followed by high-dose chemotherapy and stem cell transplant and relapsed while on or after their last line of therapy, or patients who had received multi-agent systemic chemotherapy as the 1^st^ line chemotherapy and at least one line of multi-agent systemic chemotherapy in subsequent lines of chemotherapy. We also included patients with refractory cHL, defined as failure to achieve CR after at least 4 cycles of treatment, or PR after at least 2 cycles of treatment, or progressive disease (PD) as the best objective response (BOR) or the cause of discontinued medication on any number of cycles of treatment.

Other inclusion criteria were 1) an Eastern Cooperative Oncology Group (ECOG) performance status score of 0 or 1; 2) life expectancy ≥ 3 months; 3) at least one measurable lesion (>1.5 cm in the longest diameter, or >1 cm in the longest diameter with uptake on ^18^FDG-PET) according to the 2014 Lugano criteria; 4) adequate organ function. The main exclusion criteria were 1) nodular lymphocyte-predominant HL or gray zone lymphoma; 2) active immune disease and inflammatory bowel disease; 3) central nervous system (CNS) involvement; 4) receipt of allogeneic organ transplant and allogeneic hematopoietic stem cell transplant, or receipt of ASCT within 90 days of first penpulimab dose; 5) receipt of prednisone (>10 mg/day or equivalent) or immune modulators for at least 2 weeks prior to the start of penpulimab therapy; 6) receipt of concurrent chemotherapy, immunotherapy, biological therapy or hormone therapy; 7) receipt of any anti-programmed death 1 (PD-1), anti-PD-1 ligands 1 (PD-L1) or anti-cytotoxic T-lymphocyte antigen 4 (CTLA4) antibodies, or any other antibody or drug targeting T-cell costimulation or checkpoint pathways; 8) known allergy to penpulimab or its excipient, or antibodies of murine or human origin. We also excluded pregnant or lactating women. Detailed eligibility requirements are described in Supplementary Methods.

The study protocol was approved by the ethics committee of all participating institutions and conducted in accordance with GCP and the Declaration of Helsinki. The trial is registered with clinicaltrials.gov (NCT03722147).

### Therapeutic Intervention

The study had an 8-week safety run-in period (Part A). Three patients were infused with 200 mg penpulimab once every 2 weeks and assessed for dose-limiting toxicities (DLTs) 28 days after receipt of the first penpulimab infusion. DLTs were graded and recorded according to NCI CTCAE version 4.03 and defined as any grade 3 or above treatment-emergent toxicities. If DLTs occurred in no more than one patient during the first 28-day treatment cycle, 3 additional patients were infused with 200 mg penpulimab once every 2 weeks and assessed for DLTs. If DLTs occurred in no more than one patient among the 6 evaluable patients during the safety run-in evaluation, patients started to receive 200 mg penpulimab (infusion over 60 min) once every 2 weeks for a maximum of 24 months (Part B). Penpulimab was administered until disease progression, unacceptable toxicities, withdrawal of consent, or investigator decision per protocol, for a maximum of 24 months. At the initial PD, penpulimab was continued until confirmation of PD per Lugano 2014 criteria, absence of continuous clinical benefit judged by the investigator. Other types of anti-tumor therapy were not allowed until PD was confirmed. Supportive care was provided at the discretion of the investigators. Patients with PD on initial evaluation were allowed to continue treatment if cHL did not rapidly progress, and the patients were judged to continue to benefit from and could tolerate penpulimab therapy and had stable ECOG performance status scores, and penpulimab therapy did not interfere with treatment of severe complications such as CNS metastasis.

### Patient Evaluation

Patient demographics including age, gender, and ethnicity, previous history including history of cancer and cancer treatment were recorded. Responses were evaluated by an independent radiology review committee (IRRC) per the 2014 Lugano classification based on contrast-enhanced computed tomography (CT) or fluorodeoxyglucose positron emission tomography (PET)-CT scans. Contrast-enhanced CT scans were conducted at baseline and weeks 8, 16, and 24 for the first 24 weeks and every 12 weeks thereafter and every 16 weeks after week 48 until initiation of new antitumor therapy, PD, death, or withdrawal of consent. PET-CT was conducted at baseline and weeks 8, 16, and 24, and when CR or PD was determined by CT.

The patients were followed up every three months upon completion of the final penpulimab dose. Survival was evaluated every three months. The primary efficacy outcome was ORR per the 2014 Lugano classification, which was the proportion of patients whose BOR was CR or PR. Secondary efficacy outcomes included duration of response (DoR), disease control rate (DCR), PFS, and time to tumor response (TTR).

### Safety Evaluation

Physical examinations including vital signs and ECOG performance status, routine blood chemistries and hematological tests, thyroid function tests for thyroid-stimulating hormone (TSH), free and total T3 and T4, and urinary tests were done on days 1 and 15 of each cycle. Urinary hCG test was done at baseline and when clinically indicated in women of reproductive age. In addition, ECG was obtained at baseline and every 2 cycles, and ultrasonography was done when clinically indicated. AEs were graded and recorded according to NCI CTCAE version 4.03 and coded using MedDRA 22.0. AEs included treatment-emergent AEs (TEAEs), TRAEs and irAEs.

### Statistical Analysis

Assuming an ORR of 35% using the null hypothesis and that the ORR would reach 55% after penpulimab treatment (α=0.05, two-sided), a sample size of 70 was required to detect a difference of 20% with a power of 92%. Considering dropout and exclusions as a result of the central pathology review, a sample size of 80 patients was used for the study. Part A enrolled 6 patients and Part B enrolled 74 patients. Efficacy outcomes were examined in the full analysis set (FAS) that included patients (1) who had received at least one dose of penpulimab, (2) who had a diagnosis of cHL confirmed by the central pathology review panel, and (3) who met the definition of R/R cHL (see details in ***Patients***). ORR and DCR and their 95% confidence interval (CI) were estimated using the Clopper Pearson method. Exploratory analyses of the heterogeneity of treatment effects among subgroups were performed. ORR and its 95% CI were presented graphically using forest plots.

AEs were calculated based on patients who had received at least one dose of penpulimab.

All statistical analyses were conducted using SAS version 9.4 (SAS Institute Inc., Cary, NC, USA).

## Results

### Patient Demographic and Baseline Characteristics

Between August 13, 2018, and November 8, 2019, the trial enrolled 94 patients from 23 study sites (Appendix I). The FAS included 85 patients (**Supplementary Figure 1**), and excluded 3 patients whose pathology was not centrally confirmed and 6 patients whose disease did not meet the definition of R/R cHL per China’s National Medical Products Administration. Patient demographic and baseline variables are described in **Table 1**. The median age was 31.9 years (range 18-67) and 61.2% patients were male. Fifty-eight (68.2%) patients had nodular sclerosing cHL and 37.6% had B symptoms. Seventy-one (83.5%) patients had stage III or IV cHL and 24.7% patients had bone marrow infiltration. The median duration from the initial diagnosis to the first penpulimab dose was 24.2 months. All patients had received prior chemotherapy with a median of 3 prior lines of chemotherapy (range 2-11), and 52.9% patients had received ≥3 lines of chemotherapy. Of note, 40 patients received 2 prior lines of chemotherapy and their characteristics are shown in **Supplementary Table 1**. In addition, 16.5% patients had received ASCT, and 48.2% patients had received prior radiotherapy. No patients received brentuximab vedotin.

**Table 1:** Patient demographic and baseline characteristics.

Variables	FAS (N=85)
**Age (y), median (range)**	31.9 (18, 67)
**Male sex**	52 (61.2)
**ECOG performance score**	
0	63 (74.1)
1	22 (25.9)
**B symptoms**	32 (37.6)
**Clinical stage**	
I	1 (1.2)
II	13 (15.3)
III	16 (18.8)
IV	55 (64.7)
**Histology**	
Nodular sclerosing Hodgkin lymphoma (NSHL)	58 (68.2)
Mixed cellularity (MCHL)	20 (23.5)
Lymphocyte-rich classical HL (LRCHL)	4 (4.7)
Unknown	3 (3.5)
**Duration from initial diagnosis, median (range)**	24.2 (3.4, 290.7)
**Bone marrow infiltration**	21 (24.7)
**Previous chemotherapy**	
Median (range) lines	3 (2, 11)
≥3 lines	45 (52.9)
**Other previous therapies**	
Surgery	7 (8.2)
Radiotherapy	41 (48.2)
Autologous hematopoietic stem cell transplant	14 (16.5)
Brentuximab vedotin	0

Data are shown as n (%) unless indicated otherwise.

### Efficacy Measures

**Figure 1:**
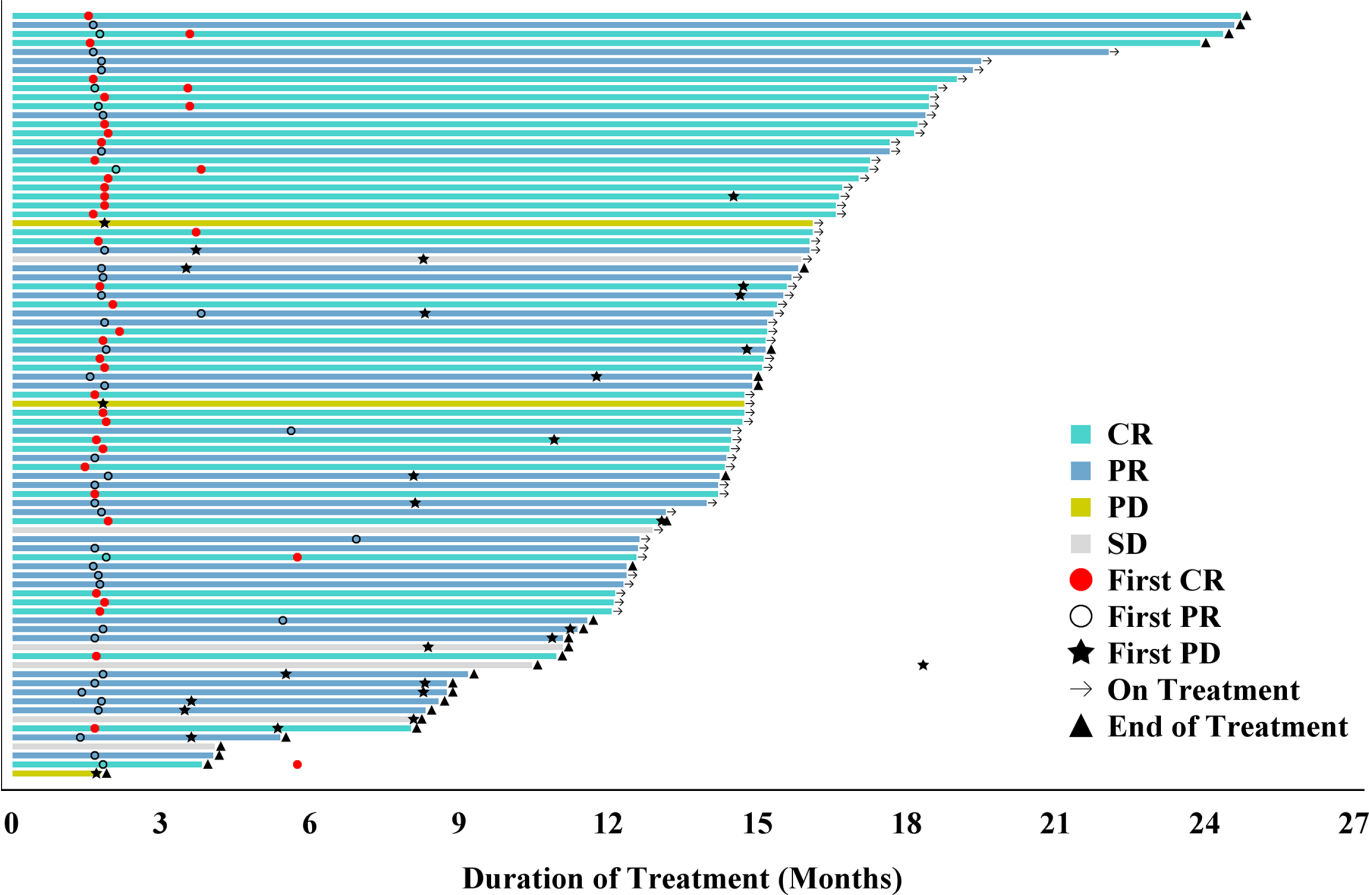
Swimmer plot of time to tumor response (months) of relapsed/refractory classical Hodgkin lymphoma patients receiving penpulimab. Responses were evaluated by the Independent Radiology Review Committee (IRRC) according to the 2014 Lugano classification. Each bar represents one patient in the full analysis set (FAS). CR, complete response; NA, not applicable; PD, progressive disease; PR, partial response; SD, stable disease.

**Figure 2:**
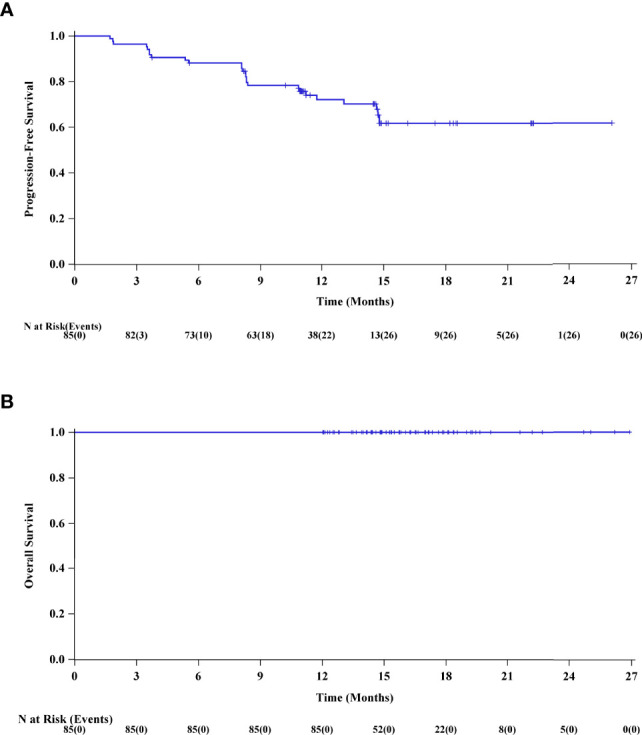
**(A)** The Kaplan-Meier curve of progression-free survival (PFS) of the FAS. **(B)** The Kaplan-Meier curve of overall survival (OS) of the FAS.

The median duration of follow-up was 15.8 months (range 12.1-26.9). Based on the review by the IRRC, 40 (47.1%) patients achieved CR, 36 (42.4%) patients achieved PR, and 6 (7.1%) patients had SD. The ORR was 89.4% (95% CI 80.8%, 95.0%), and the DCR was 96.5% (95% CI 90.0%, 99.3%). At the last follow-up, 73.7% patients remained in remission. The median TTR was 1.8 months (range 1.4, 6.9). DoR lasted for 12 months in 74.9% (95% CI: 62.4%, 83.8%) patients and the median DoR was not yet reached (**Table 2**). The duration of treatment and TTR of individual patients as well as other details including time to first CR, PR, or PD are shown in the swimmer plot (**Figure 1**). The 6 and 12-month PFS rate was 88.2% (95% CI: 79.2%, 93.5%) and 72.1% (95% CI 60.5%, 80.8%), respectively. The median PFS was not reached (**Figure 2A**). The median OS was also not reached, and the 18-month OS rate was 100% (**Figure 2B**).

**Table 2:** Best objective response by the 2014 Lugano classification based on IRC evaluation of the full analysis set (FAS; n=85).

	IRC-evaluated
**ORR, n (%) (95% CI)**	76 (89.4) (80.8, 95.0)
**DCR, n (%) (95% CI)**	82 (96.5) (90.0, 99.3)
CR, n (%)	40 (47.1)
PR, n (%)	36 (42.4)
SD, n (%)	6 (7.1)
PD, n (%)	3 (3.5)
Not evaluable, n (%)	0
**TTR (month), median (range)**	1.8 (1.4, 6.9)
**DoR (month)^[a]^**	
Median (range)	NR (1.7, 24.5+) ^b^
95% CI	13.0, NE
6	89.4 (80.0, 94.6)
9	83.8 (73.2, 90.5)
12	74.9 (62.4, 83.8)
Still remaining in response, n (%) ^[a]^	56 (73.7)
**PFS (month) ^a^**	
Median	NR (NE, NE)
6	88.2 (79.2, 93.5)
9	78.4 (67.9, 85.8)
12	72.1 (60.5, 80.8)
**OS (month) ^a^**	
Median	NR (NE, NE)
12	100 (100, 100)
18	100 (100, 100)

CI, confidence interval; CR, complete response; DoR, duration of response; IRC: Independent Review Committee; NE, not evaluable; NR, not reached; ORR, objective response rate; OS, overall survival; PFS, progression-free survival; PR, partial response; SD, stable disease; TT, time to tumor response. ^a^ Kaplan-Meier method; ^b^ some cases were censored.

**Figure 3:**
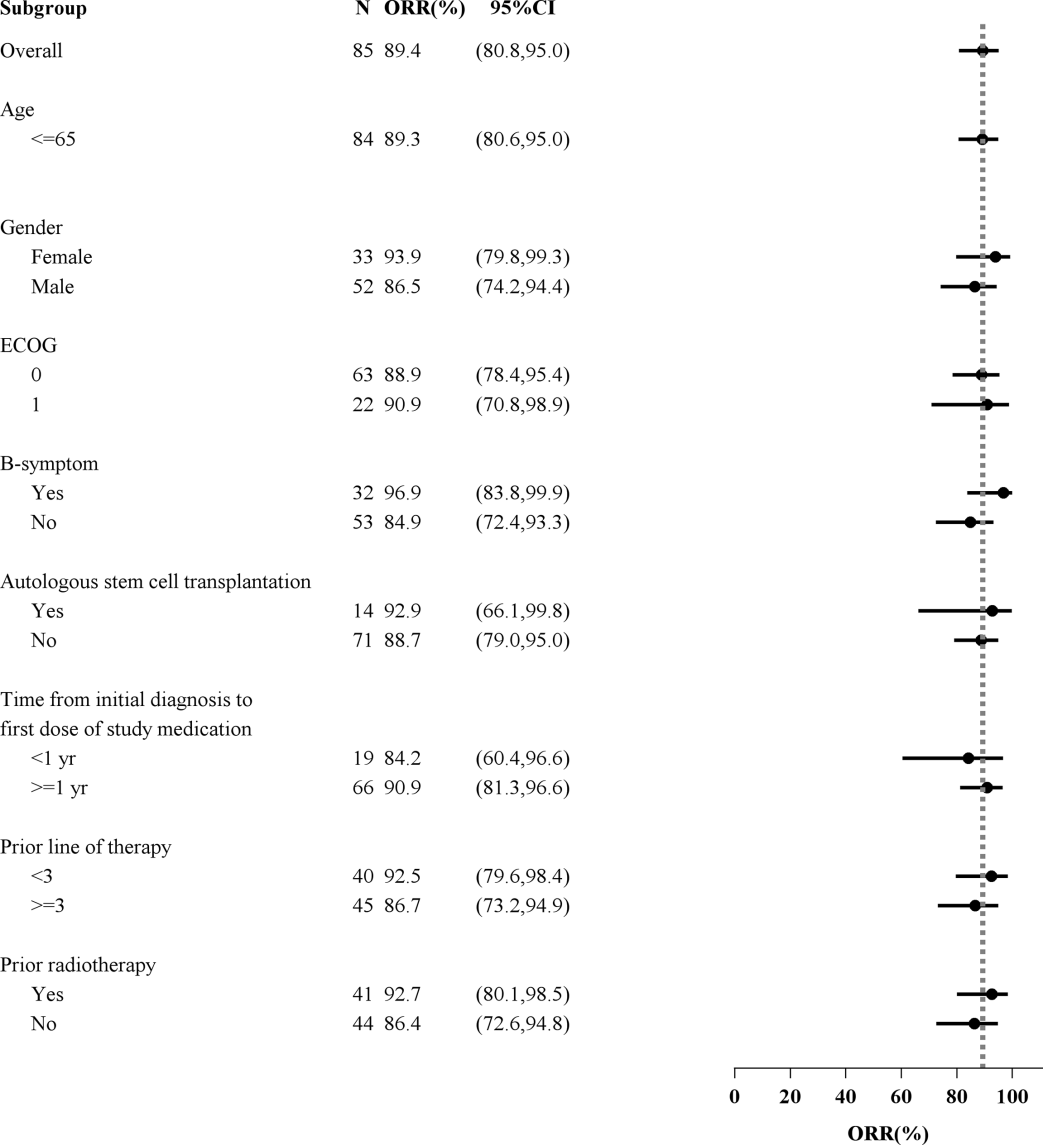
Forest plot analysis of objective response rate (ORR) by patient subgroups.

The subgroup analysis showed no significant difference in the ORR between any subgroups based on age (≤65 or >65 years), sex, ECOG performance status score (0 or 1), B symptoms, previous lines of therapy (<3 or ≥3), time from initial diagnosis to the first dose (<1 or ≥1 years), previous radiotherapy and prior ASCT (**Figure 3**).

### Safety

#### TRAEs

The median total exposure time was 449 days (range 55, 753) and 96.8%, 91.5%, and 76.6% patients had total exposure time ≥3, 6, and 12 months, respectively. The median number of penpulimab infusions was 28.0 (IQR 21.0, 33.0) (**Supplementary Table 2**). Sixty (63.8%) patients in 94 eligible patients were still receiving penpulimab therapy at the cut-off date.

TEAEs and TRAEs (including unlikely related) with an incidence ≥10% and CTCAE grade 3 and above TEAEs and TRAEs are shown in **Table 3**. Ninety-two (97.9%) patients experienced at least one TRAE. TRAEs with an incidence ≥ 20% included hypothyroidism (31.9%), upper respiratory tract infection (25.5%), and fever (24.5%). The incidence of CTCAE grade 3 and above TRAEs was 26.6%, including reduced platelet count, hyperlipidemia, and skin rash each in 3 (3.2%) patients, and reduced neutrophil count in 2 (2.1%) patients. Serious adverse events (SAEs) were reported in twelve (12.8%) patients. Ten (10.6%) patients developed severe TRAEs. Penpulimab was interrupted in 21.3% of patients due to TRAEs, including leucopenia and hypothyroidism each in 2 (2.1%) patients. Penpulimab was discontinued in 5 (5.3%) patients, one due to hemophagocytic lymphohistiocytosis and the remaining four due to irAEs (see below for details). One patient died from hemophagocytic lymphohistiocytosis.

**Table 3:** TEAEs and TRAEs (with an incidence ≥10%) and CTCAE grade 3 and above TEAEs and TRAEs in the safety set (n=95).

Events, n (%)	Treatment-emergent		Treatment-related	
	All grades (≥10%)	Grade III and above	All grades (≥10%)	Grade III and above
Upper respiratory tract infection	35 (37.2)	0	24 (25.5)	0
Hypothyroidism	30 (31.9)	0	30 (31.9)	0
Body weight gain	28 (29.8)	3 (3.2)	17 (18.1)	1 (1.1)
Fever	23 (24.5)	1 (1.1)	23 (24.5)	1 (1.1)
Hyperuricemia	23 (24.5)	2 (2.1)	17 (18.1)	0
Elevated ALT	22 (23.4)	0	22 (23.4)	0
Hypertriglyceridemia	20 (21.3)	1 (1.1)	18 (19.1)	1 (1.1)
Reduced leucocyte count	18 (19.1)	1 (1.1)	17 (18.1)	1 (1.1)
Skin rash	16 (17.0)	3 (3.2)	15 (16.0)	3 (3.2)
Elevated AST	15 (16.0)	0	14 (14.9)	0
Anemia	14 (14.9)	1 (1.1)	14 (14.9)	1 (1.1)
Elevated TSH	13 (13.8)	0	13 (13.8)	0
Reduced neutrophil count	13 (13.8)	2 (2.1)	12 (12.8)	2 (2.1)
Hyperlipidemia	11 (11.7)	3 (3.2)	6 (6.4)	3 (3.2)
Back pain	11 (11.7)	0	9 (9.6)	0
Hyperthyroidism	11 (11.7)	0	10 (10.6)	0
Transfusion-related reaction	11 (11.7)	0	11 (11.7)	0
Diarrhea	11 (11.7)	0	8 (8.5)	0
Urinary tract infections	11 (11.7)	0	8 (8.5)	0
Body weight loss	10 (10.6)	0	8 (8.5)	0
Leucopenia	10 (10.6)	0	9 (9.6)	0

ALT, alanine transaminase; AST, aspartate aminotransferase; TSH, thyroid-stimulating hormone.

#### IrAEs

Fifty-one (54.3%) patients experienced an irAE, but only 4 (4.3%) patients developed grade 3 and above irAEs (**Supplementary Table 3**). The three most frequent irAEs included hypothyroidism (28.7%), TSH elevations (10.6%), and hyperthyroidism (6.4%). When thyroid-related AEs were excluded, the incidence of irAEs was 17.0%. Grade 3 irAEs occurred in 4 (4.3%) patients, including kidney injury, immune pneumonitis, rash, and psoriasis each in 1 (1.1%) case. No grade 4 or 5 irAEs were reported. Three (3.2%) patients developed severe irAEs and 8 (8.5%) patients experienced treatment-interruptions due to irAEs. Four (4.3%) patients discontinued treatment due to immune-related pneumonitis (n=2), mesangial proliferative glomerulonephritis (n=1) and IgA nephropathy (n=1). No death due to irAE was reported.

## Discussion

In this single arm phase II trial, R/R cHL patients were treated with penpulimab (200 mg Q2W). The dosing regimen was mainly based on the results of an earlier phase Ia study (AK105-101) showing that penpulimab at a dose of 3 mg/kg body weight could saturate peripheral PD-1 rapidly (within 24 h) and maintain an occupancy rate above 90% for PD-1 at day 15 (before the second dose). Based on the safety and pharmacokinetics (PK) data of the phase I study, a dose of 200 mg penpulimab (equivalent to 3.3 mg/kg body weight in a person with a body weight of 60 kg) was recommended for the phase II trial.

In this trial, the ORR with penpulimab was 89.4%. The rate of CR and PR was 47.1% and 42.4%, respectively. Approximately three quarters (73.7%) of the patients remained in remission after a median follow-up of 15.8 months. The 12-month PFS rate was 72.1% and the 18-month OS rate was 100%. The treatment benefit was seen across all patient subgroups including previous lines of therapy (<3 or ≥3) and ASCT (yes or no). When compared with historical controls, this study showed that penpulimab as a single agent is more effective than traditional chemotherapies in the third line and above setting (22, 23). Moreover, penpulimab exhibited a safety profile and efficacy comparable to or better than those of other PD-1 antibodies (8, 10, 12, 24). The incidence of grade 3 and above irAEs was low (4.3%) and no death due to irAEs occurred. There was also a lower incidence of SAEs (12.8%) than previous reports (15% - 26%) (6, 10, 12, 25). Though one patient died of hemophagocytic lymphohistiocytosis, the death was deemed unlikely to be related to penpulimab treatment by the investigator. Secondary lung infection or recurrence of lymphoma was more likely the cause of death. The rate of transfusion reaction (11.7%) and fever (24.5%) was lower with penpulimab *versus* other anti-PD-1 monoclonal bodies for Chinese R/R cHL patients (tislelizumab: 38.6% and 54.3%; sintilimab: 12% and 46%; camrelizumab: 16.0% and 44.0%) (8, 10, 12). In addition, a study summarizing the safety data of penpulimab from 6 clinical studies involving a total of 465 patients with solid tumors and lymphoma revealed that penpulimab had good safety and tolerability. Specifically, the incidence of irAEs was 27.1% and that of CTCAE grade 3 irAEs was 3.2%, which were immune-related hepatitis (0.6%), immune-related skin adverse reaction (1.3%), immune-related pneumonitis (0.6%), immune-related hypophysitis (0.4%), and immune-related nephritis [kidney injury (IgA nephropathy)], 0.2%. No CTCAE grade 4 or 5 irAEs were reported (manuscript under preparation).

The mechanisms underlying the development of irAEs are not fully clarified and one possible reason is the production of cytokines such as IL-6 and TNF-α induced through Fc-binding (16–18). Fc mutation was introduced into penpulimab, which effectively eliminated FcR-mediated effector function and remarkably abated inflammatory cytokine release from both non-activated and activated PBMCs, hinting at a benign safety profile in terms of irAE occurrences. Meanwhile, elimination of FcR-mediated effector function could avoid ADCC, CDC and ADCP-mediated T cell injury, which is conducive to the enhancement of the efficacy of anti-PD-1 monoclonal antibodies (19).

The better efficacy and safety profile of penpulimab could also be attributed to its IgG1 antibody subtype, which has a stable structure and avoids tumor cell immune escape as observed with IgG4 antibodies. Recent studies have found that IgG4 plays a crucial role in coordinating an inhibitory effect of humoral and cellular immune responses against cancer (26). IgG4 antibody has unique structural features that make it possible to bind to other IgGs, in particular IgG1, *via* Fc-Fc interactions (27). IgG4, regardless of its antigen specificity, inhibits the classic immune reactions of cancer-specific IgG1 against cancer cells *in vitro*. It was also found that anti-PD-1 antibody of IgG4 subtype significantly promoted cancer growth in mice (14).

This trial has several limitations. First, this is a single-arm trial and OS was not used as the primary outcome. In addition, the duration of follow-up was short (median, 15.8 months). These findings need to be validated in randomized controlled studies with long term follow-up. Second, no subjects had received prior therapy with brentuximab vedotin as the drug had not been approved for CD30-positive lymphoma in China at the time of this trial. Another limitation of this study is that only 16.5% patients had received ASCT, which is in line with the low rate of ASCT in cHL patients across China and comparable to the rate of ASCT for tislelizumab (18.6%), sintilimab (19%), and camrelizumab (12%) (8, 10, 12).

Currently, monotherapy with brentuximab vedotin or anti-PD-1 monoclonal antibodies remain the predominant treatment for R/R cHL. Brentuximab vedotin was not yet approved in China at the time of this trial. There was also no data on pembrolizumab and nivolumab or any other anti-PD-1 monoclonal antibodies for Chinese cHL patients and clinical access to these agents remained extremely limited. The current findings are of clinical relevance in China and resource-constrained countries. Earlier studies have shown that anti-PD-1 monoclonal antibody monotherapy has noticeable efficacy in R/R cHL patients who have failed multiple lines of chemotherapy, which prompted us to pursue a single arm design for the current trial, with ORR being the primary efficacy measure. One feature of immunotherapy is the remarkable durability of remission once it has been achieved. We will report the longer term results of penpulimab treatment for R/R cHL when they become available, which will shed further light on the efficacy and safety of penpulimab in this patient population. Meanwhile, we are conducting confirmatory phase III trials to validate the findings of the current study. In conclusion, the novel anti-PD-1 antibody penpulimab achieved a high ORR, long survival with an acceptable safety profile in this trial, providing an important efficacious and safe treatment option for Chinese R/R cHL patients.

## Data Availability Statement

The raw data supporting the conclusions of this article will be made available by the authors, without undue reservation.

## Ethics Statement

The studies involving human participants were reviewed and approved by Peking University Cancer Hospital & Institute; The Affiliated Cancer Hospital of Zhengzhou University and Henan Cancer Hospital; Cancer Hospital Affiliated to Guangzhou Medical University Tianjin Medical University Cancer Institute and Hospital; National Clinical Research Center of Cancer, Key Laboratory of Cancer Prevention and Therapy, the Sino-US Center for Lymphoma and Leukemia Research; Qilu Hospital, Shandong University; The First Affiliated Hospital with Nanjing Medical University, Jiangsu Province Hospital, Collaborative Innovation Center for Cancer Personalized Medicine; The First Affiliated Hospital of Nanchang University; The First Affiliated Hospital of USTC, Division of Life Sciences and Medicine, University of Science and Technology of China Tumor Hospital of Xiangya School of Medicine of Central South University; Liaoning Cancer Hospital and Institute; Nanjing Drum Tower Hospital, Clinical College of Nanjing Medical University; Second Affiliated Hospital of Dalian Medical University; Shanghai First People’s Hospital, Shanghai Jiaotong University; West China Hospital, Sichuan University; Heilongjiang Provincial Hospital; The Fourth Hospital of Hebei Medical University; Weifang People’s Hospital; Union Hospital, Tongji Medical College, Huazhong University of Science and Technology; Shaanxi Provincial Cancer Hospital; Shanghai Tongren Hospital; The Affiliated Hospital of Qingdao University; The First Affiliated Hospital of Guangxi Medical University; The Third Affiliated Hospital of Suzhou University, The First People’s Hospital of Changzhou. The patients/participants provided their written informed consent to participate in this study.

## Author Contributions

Conception and design: JZ, YS, YX, BL; Development of methodology: JZ, YS, XJ, MZ; Acquisition of data: YS, KZ, CJ, ZQ, MH, LF, FL, KD, HZ, XL, BC, XHS, XMS, MJ, QZ, LHL, GY, YH, ZZ, LGL, HX, JL, BH, JZ; Analysis and interpretation of data: YS, KZ, CJ, ZQ, MH, LF, FL, KD, HZ, XL, BC XHS, XMS, MJ, QZ, LHL, GY, YH, ZZ, LGL, HX, JL, BH, XJ, MZ, JZ; Writing, review, and/or revision of the manuscript: YS, KZ, CJ, ZQ, MH, LF, FL, KD, HZ, XL, BC, XHS, XMS, MJ, QZ, LHL, GY, YH, ZZ, LGL, HX, JL, BH, XJ, MZ, JZ; All authors reviewed and approved the final version of the manuscript.

## Funding

Funding for this study was provided by Akeso Biopharma Co., Ltd.

## Conflict of Interest

XJ, MZ, BL and YX are employees of Akeso Biopharma Co., Ltd.

The remaining authors declare that the research was conducted in the absence of any commercial or financial relationships that could be construed as a potential conflict of interest.

The authors declare that this study received funding from Akeso Biopharma Co., Ltd. The funder had the following involvement with the study: sponsored this trial, and participated in the study design, data collection, statistical analysis, and provided medical writing support.

## Publisher’s Note

All claims expressed in this article are solely those of the authors and do not necessarily represent those of their affiliated organizations, or those of the publisher, the editors and the reviewers. Any product that may be evaluated in this article, or claim that may be made by its manufacturer, is not guaranteed or endorsed by the publisher.

## References

[1] Liu W Liu J Song Y Wang X Zhou M Wang L. Mortality of Lymphoma and Myeloma in China, 2004-2017: An Observational Study. J Hematol Oncol (2019) 12:22. doi: 10.1186/s13045-019-0706-9 30832702 PMC6399942

[2] Ansell SM. Hodgkin Lymphoma: 2018 Update on Diagnosis, Risk-Stratification, and Management. Am J Hematol (2018) 93:704–15. doi: 10.1002/ajh.25071 29634090

[3] Kuruvilla J Ramchandren R Santoro A Paszkiewicz-Kozik E Gasiorowski R Johnson NA. Pembrolizumab Versus Brentuximab Vedotin in Relapsed or Refractory Classical Hodgkin Lymphoma (KEYNOTE-204): An Interim Analysis of a Multicentre, Randomised, Open-Label, Phase 3 Study. Lancet Oncol (2021) 22:512–24. doi: 10.1016/S1470-2045(21)00005-X 33721562

[4] Younes A Santoro A Shipp M Zinzani PL Timmerman JM Ansell S. Nivolumab for Classical Hodgkin’s Lymphoma After Failure of Both Autologous Stem-Cell Transplantation and Brentuximab Vedotin: A Multicentre, Multicohort, Single-Arm Phase 2 Trial. Lancet Oncol (2016) 17:1283–94. doi: 10.1016/S1470-2045(16)30167-X PMC5541855 27451390

[5] Armand P Shipp MA Ribrag V Michot JM Zinzani PL Kuruvilla J. Programmed Death-1 Blockade With Pembrolizumab in Patients With Classical Hodgkin Lymphoma After Vrentuximab Vedotin Failure. J Clin Oncol (2016) 34:3733–9. doi: 10.1200/JCO.2016.67.3467 PMC5791838 27354476

[6] Ansell SM Lesokhin AM Borrello I Halwani A Scott EC Gutierrez M. PD-1 Blockade With Nivolumab in Relapsed or Refractory Hodgkin’s Lymphoma. N Engl J Med (2015) 372:311–9. doi: 10.1056/NEJMoa1411087 PMC4348009 25482239

[7] Chen R Zinzani PL Fanale MA Armand P Johnson NA Brice P. Phase II Study of the Efficacy and Safety of Pembrolizumab for Relapsed/Refractory Classic Hodgkin Lymphoma. J Clin Oncol (2017) 35:2125–32. doi: 10.1200/JCO.2016.72.1316 PMC5791843 28441111

[8] Song Y Gao Q Zhang H Fan L Zhou J Zou D. Treatment of Relapsed or Refractory Classical Hodgkin Lymphoma With the Anti-PD-1, Tislelizumab: Results of a Phase 2, Single-Arm, Multicenter Study. Leukemia (2020) 34:533–42.x. doi: 10.1038/s41375-019-0545-2 31520078 PMC7214259

[9] Lee A Keam SJ. Tislelizumab: First Approval. Drugs (2020) 80:617–24. doi: 10.1007/s40265-020-01286-z 32185681

[10] Shi Y Su H Song Y Jiang W Sun X Qian W. Safety and Activity of Sintilimab in Patients With Relapsed or Refractory Classical Hodgkin Lymphoma (ORIENT-1): A Multicentre, Single-Arm, Phase 2 Trial. Lancet Haematol (2019) 6:e12–9. doi: 10.1016/S2352-3026(18)30192-3 30612710

[11] Hoy SM. Sintilimab: First Global Approval. Drugs (2019) 79:341–6. doi: 10.1007/s40265-019-1066-z 30742278

[12] Wu J Song Y Chen X Lin T Cao J Liu Y. Camrelizumab for Relapsed or Refractory Classical Hodgkin Lymphoma: Extended Follow-Up of the Multicenter, Single-Arm, Phase 2 Study. Int J Cancer (2022) 150:984–92. doi: 10.1002/ijc.33852 34674396

[13] Markham A Keam SJ. Camrelizumab: First Global Approval. Drugs (2019) 79:1355–61. doi: 10.1007/s40265-019-01167-0 31313098

[14] Wang H Xu Q Zhao C Zhu Z Zhu X Zhou J. An Immune Evasion Mechanism With IgG4 Playing an Essential Role in Cancer and Implication for Immunotherapy. J Immunother Cancer (2020) 8:e000661. doi: 10.1136/jitc-2020-000661 32819973 PMC7443307

[15] Zhang T Song X Xu L Ma J Zhang Y Gong W. The Binding of an Anti-PD-1 Antibody to FcγRI Has a Profound Impact on Its Biological Functions. Cancer Immunol Immunother (2018) 67:1079–90. doi: 10.1007/s00262-018-2160-x PMC6006217 29687231

[16] Tanaka R Okiyama N Okune M Ishitsuka Y Watanabe R Furuta J. Serum Level of Interleukin-6 is Increased in Nivolumab-Associated Psoriasiform Dermatitis and Tumor Necrosis Factor-α Is a Biomarker of Nivolumab Recativity. J Dermatol Sci (2017) 86:71–3. doi: 10.1016/j.jdermsci.2016.12.019 28069323

[17] Rotz SJ Leino D Szabo S Mangino JL Turpin BK Pressey JG. Severe Cytokine Release Syndrome in a Patient Receiving PD-1-Directed Therapy. Pediatr Blood Cancer (2017) 12:64. doi: 10.1002/pbc.26642 28544595

[18] Kinder M Greenplate AR Strohl WR Jordan RE Brezski RJ. An Fc Engineering Approach That Modulates Antibody-Dependent Cytokine Release Without Altering Cell-Killing Functions. MAbs (2015) 7:494–504. doi: 10.1080/19420862.2015.1022692 25933349 PMC4622058

[19] Li B Huang Z Pang X Zhong T Jin C Chen N. 2o Penpulimab, an IgG1 Anti-PD-1 Antibody With Fc-Engineering to Eliminate Effector Functions and With Unique Epitope and Binding Properties. Ann Oncol (2021) 32:S361. doi: 10.1016/j.annonc.2021.08.280

[20] Tiller KE Tessier PM. Advances in Antibody Design. Annu Rev BioMed Eng (2015) 17:191–216. doi: 10.1146/annurev-bioeng-071114-040733 26274600 PMC5289076

[21] Ito T Tsumoto K. Effects of Subclass Change on the Structural Stability of Chimeric, Humanized, and Human Antibodies Under Thermal Stress. Protein Sci (2013) 22:1542–51. doi: 10.1002/pro.2340 PMC3831669 23963869

[22] Arai S Fanale M DeVos S Engert A Illidge T Borchmann P. Defining a Hodgkin Lymphoma Population for Novel Therapeutics After Relapse From Autologous Hematopoietic Cell Transplant. Leuk Lymphoma (2013) 54:2531–3. doi: 10.3109/10428194.2013.798868 23617324

[23] Schmitz N Pfistner B Sextro M Sieber M Carella AM Haenel M. Aggressive Conventional Chemotherapy Compared With High-Dose Chemotherapy With Autologous Haemopoietic Stem-Cell Transplantation for Relapsed Chemosensitive Hodgkin’s Disease: A Randmised Trial. Lancet (2002) 359:2065–71. doi: 10.1016/S0140-6736(02)08938-9 12086759

[24] Chen R Zinzani PL Lee HJ Armand P Johnson NA Brice P. Pembrolizumab in Relapsed or Refractory Hodgkin Lymphoma: 2-Year Follow-Up of KEYNOTE-087. Blood (2019) 134:1144–53. doi: 10.1182/blood.2019000324 PMC6776792 31409671

[25] Song Y Gao Q Zhang H Fan L Zhou J Zou D. Tislelizumab for Relapsed/Refractory Classical Hodgkin Lymphoma: 3-Year Follow-Up and Correlative Biomarker Analysis. Clin Cancer Res (2021) 6:1147–56. doi: 10.1158/1078-0432.CCR-21-2023 PMC9365351 34716199

[26] Bianchini R Karagiannis SN Jordakieva G Jensen-Jarolim E. The Role of IgG4 in the Fine Tuning of Tolerance in IgE-Mediated Allergy and Cancer. Int J Mol Sci (2020) 21:5017. doi: 10.3390/ijms21145017 PMC7404042 32708690

[27] Crescioli S Correa I Karagiannis P Davies AM Sutton BJ Nestle FO. IgG4 Characteristics and Functions in Cancer Immunity. Curr Allergy Asthma Rep (2016) 16:7. doi: 10.1007/s11882-015-0580-7 26742760 PMC4705142

