# Supplementary material for: Penpulimab for Relapsed or Refractory Classical Hodgkin Lymphoma: A Multicenter, Single-Arm, Pivotal Phase I/II Trial (AK105-201)
Source: Front Oncol. 2022 Jul 7;12:925236. doi: 10.3389/fonc.2022.925236 (PMC9301139; doi:10.3389/fonc.2022.925236)
Supplement: Supplementary file 1 [file DataSheet_1.docx]

**Supplementary Methods**

Patient eligibilities

Inclusion Criteria

1. Written and signed informed consent.
2. Eastern Cooperative Oncology Group (ECOG) Performance Score of 0 or 1.
3. Histologically confirmed classical Hodgkin's lymphoma (cHL) (based on tumor tissue obtained within 3 years prior to enrollment).
4. Relapsed (disease progression during or after most recent therapy) or refractory (failure to achieve CR or PR after most recent therapy) cHL and meet any of the following criterions:
   1. Recurrence or disease progression after autologous hematopoietic stem cell transplantation.
   2. For subject without receiving ASCT, the subject has received at least 2 lines of prior systemic chemotherapy. Refractory subject is defined as subject who has not achieved PR after at least 2 cycles of treatment, or subject who has not achieved CR after at least 4 cycles of treatment. If the best response to treatment is PD or the reason for ending the treatment is PD, the subject is considered as refractory without requirement on the number of cycles of treatment that the subject has received. For relapsed patients, disease progression occurred for the subject who has received at least 2 lines of prior systemic chemotherapy.
5. Subject must have at least one measurable lesion (> 1.5 cm in the longest diameter, or > 1 cm in the longest diameter with uptake on ^18^FDG-PET) according to the Lugano 2014 criteria.
6. Adequate organ functions.

Exclusion Criteria

1. Known nodular lymphoma predominant Hodgkin lymphoma or Grey zone lymphoma.
2. Lymphoma involving the central nervous system.
3. Participated in other clinical studies of experimental drugs or received research treatment or used experimental equipment within 4 weeks prior to the first dose of penpulimab.
4. Concurrent enrollment in another clinical study, unless it is an observational (non-interventional) clinical study or the follow-up period of an interventional study.
5. Receipt of the last radiotherapy or the last dose of anticancer therapy (chemotherapy, target therapy, immunotherapy, or tumor embolism, etc.) with 4 weeks prior to the first dose of penpulimab. Receipt of the last dose of nitrocarbamide or mitomycin C within 6 weeks prior to the first dose of penpulimab.
6. Prior exposure to any anti-PD-1, anti-PD-L1, anti-CTL4 antibody or any other antibody or drug targeting T-cell costimulation or checkpoint pathways such as ICOS, or agonists such as CD40, CD137, GITR, OX40 etc..
7. Had other active malignancies within 5 years prior to enrollment. Locally curable cancer (manifested as cured) is excluded, such as basal or cutaneous squamous cell carcinoma, superficial bladder cancer, cervical or breast carcinoma in situ.
8. Active, known or suspected autoimmune diseases, or a history of the disease within the past 2 years, except the following: vitiligo, alopecia, Graves' disease, psoriasis or eczema that do not require systemic treatment within the last 2 years, hypothyroidism (caused by autoimmune thyroiditis) only requiring a stable dose of hormone replacement therapy, type I diabetes requiring only a stable dose of insulin replacement therapy, or diseases not expected to recur in the absence of external triggering factors.
9. Active or prior documented inflammatory bowel disease (e.g., Crohn's disease, ulcerative colitis or chronic diarrhea).
10. patients with a condition requiring systemic treatment with either corticosteroid (> 10 mg daily prednisone equivalents) or other immunosuppressive medications within 14 days of study drug administration.
11. History of testing positive for human immunodeficiency virus (HIV) or known acquired immunodeficiency syndrome (AIDS).
12. History of primary immunodeficiency.
13. History of active tuberculosis.
14. History of allogeneic stem cell transplantation or organ transplantation.
15. Autologous hematopoietic stem cell transplantation performed within 90 days prior to the first dose of penpulimab.
16. History of gastrointestinal perforation and /or within 6 months prior to enrollment.
17. History of interstitial lung disease.
18. Patients with untreated chronic hepatitis B or with HBV DNA exceeding 500 IU/mL, or with active hepatitis C should be excluded. Inactive HBsAg carriers, treated and stable hepatitis B patients (HBV DNA < 500 IU/mL), or cured hepatitis C patients can be enrolled. For patients with positive HCV antibody, they are eligible to participate in the study only if the test result of HCV RNA is negative.
19. Major surgical procedure (as defined by the investigator) within 30 days prior to the first dose of penpulimab or still recovering from prior surgery. Local procedures (e.g., placement of a systemic port, core needle biopsy, and prostate biopsy) are allowed if completed at least 24 hours prior to the administration of the first dose of study treatment.
20. Uncontrolled pleural effusion, pericardial effusion, or ascites requiring repeated drainage.
21. Active infections requiring systemic treatment.
22. Uncontrolled concurrent disease, including but not limited to, persistent or active infection, symptomatic congestive heart failure (according to the New York heart association functional class defined 3 or 4), out of control of high blood pressure, unstable angina, arrhythmia, severe peptic ulcer or gastritis, activity, or mental illness/social status which will limit the participants compliance requirements or damage to the participants to provide written informed consent.
23. Unresolved toxicities from prior anticancer therapy, defined as having not resolved to National Cancer Institute's (NCI's) Common Terminology Criteria for Adverse Events (CTCAE) (NCI CTCAE v4.03) Grade 0 or 1, or to levels dictated in the inclusion/exclusion criteria with the exception of alopecia. patients with irreversible toxicity that is not reasonably expected to be exacerbated by the study drug may be included (e.g., hearing loss) after consultation with the medical monitor. patients with ≤ Grade 2 neuropathy will be evaluated on a case-by-case basis after consultation with the medical monitor.
24. Receipt of live or attenuated vaccination within 30 days prior to the first dose of penpulimab, or plan to have live or attenuated vaccination during the study.
25. Known allergy or reaction to any component of the penpulimab formulation.
26. History of severe allergic reaction to any other monoclonal antibodies.
27. Women who are pregnant or nursing.
28. Any condition that, in the opinion of the investigator, would interfere with evaluation of the investigational product or interpretation of subject safety or study results.

***Pharmacokinetics assays***

PK studies and immunogenicity assays were done at day 1 and 15 of each treatment cycle in Part A and at day 1 of each treatment cycle in Part B. Intensive sampling was done in 6 patients at day 1 of cycle 1 after the first infusion and in 5 patients at day 15 of cycle 3 after 6 infusions in Part A. In addition, 85 patients from Part B underwent sparse sampling. Four mL venous blood was drawn from each patient. Noncompartmental methods were used for calculating PK parameters of penpulimab. Plasma penpulimab concentrations were measured by ELISA. Furthermore, maximum concentration (C_max_) and time to reach C_max_ (T_max_) and clearance (CL) were calculated. The AUC_0-t_ and AUC_0-inf_ were the area under the curve (AUC) from the first to the last observation and from the first observation to the extrapolated time infinity, respectively. The PK parameters after the first penpulimab dose (day 1 of cycle 1) included the area under the blood (or plasma) concentration–time curve (AUC_[0-14d]_, AUC_[0-inf]_), C_max_ and T_max_, CL, terminal apparent volume of distribution (V_z_), steady state apparent volume of distribution (V_ss_), and mean residence time infinite (MRT_inf_), and terminal elimination half-life (t_1/2_). For convenience, AUC _(0-t)_ was replaced by AUC _(0-14d)_ in this paper. After multiple infusions (day 15 of cycle 3), the PK parameters included AUC _(0-tau),_ trough concentration (C_min, ss_), C_max, ss_, C_avg, ss_, T_max, ss_, CL, V_z_, V_ss_, MRT_inf_ and t_1/2_. The PK parameters were calculated by non-compartmental analysis (NCA) model using WinNonlin software 8.0 (Certara USA Inc, Princeton, NJ, USA), except AUC _(0-inf),_ t_1/2_, V_z_, CL, MRT_inf_, which were determined by population PK modeling.

The PK analysis set included all patients who received at least one dose of penpulimab and at least one measurable penpulimab concentration and had no major protocol violation that could interfere with measurement of penpulimab concentration. Descriptive statistics were mainly used.

**Supplementary Results**

***PK/PD properties of penpulimab***

The PK set included 91 patients. The mean C_max_ was 77.3 ± 19.9 μg/mL at the end of the first infusion and then declined to 20.4 μg/mL immediately before the second dose after slow elimination from the body (Supplementary Figure 2A). The mean AUC _(0-14d)_ of penpulimab was 11000 ± 2080 h•μg/mL. After 6 infusions, the mean Cmax was 155 ± 17.4 μg/mL and declined to 81.1 μg/mL immediately before the next dose after slow elimination (Supplementary Figure 2B). The mean accumulation ratio was 2.16 ±0.47 and 3.20 ± 0.43 based on mean C_max_ and the mean AUC, respectively, indicating that penpulimab (200 mg Q2W) accumulated to some extent after multiple infusions. The trough and peak plasma concentrations of penpulimab increased after multiple infusions until week 12 (cycle 4) or 16 (cycle 5) and remained steady thereafter (Supplementary Figure 2C).

**Supplementary Figure Legends**

**Supplementary Figure 1** The study flowchart.

**Supplementary Figure 2** (A) Linear (left panel) and semi-logarithmic (right panel) plasma concentration–time profiles of penpulimab following the first infusion of 200 mg penpulimab. (B) Linear plasma concentration–time profiles of penpulimab following multiple infusions of 200 mg penpulimab given once biweekly. (C) The mean trough and peak plasma concentrations of penpulimab in sparse sampling. CxDy, where x indicates the number of weeks and y the number of days. 0 h represents before infusion and 1 h end of infusion. C1D1 (0h) n=91; C1D1 (1h) n=91; C2D1 (0h) n=91; C2D1 (1h) n=91; C3D1 (0h) n=87; C3D1 (1h) n=87; C4D1 (0h) n=74; C4D1 (1h) n=74; C5D1 (0h) n=68; C5D1 (1h) n=68; C6D1 (0h) n=57; C6D1 (0h) n=57; C7D1 (0h) n=43; C10D1 (0h) n=19; C13D1 (0h) n=6.
