# Supplementary figures and images for: Penpulimab for Relapsed or Refractory Classical Hodgkin Lymphoma: A Multicenter, Single-Arm, Pivotal Phase I/II Trial (AK105-201)
Source: Front Oncol. 2022 Jul 7;12:925236. doi: 10.3389/fonc.2022.925236 (PMC9301139; doi:10.3389/fonc.2022.925236)

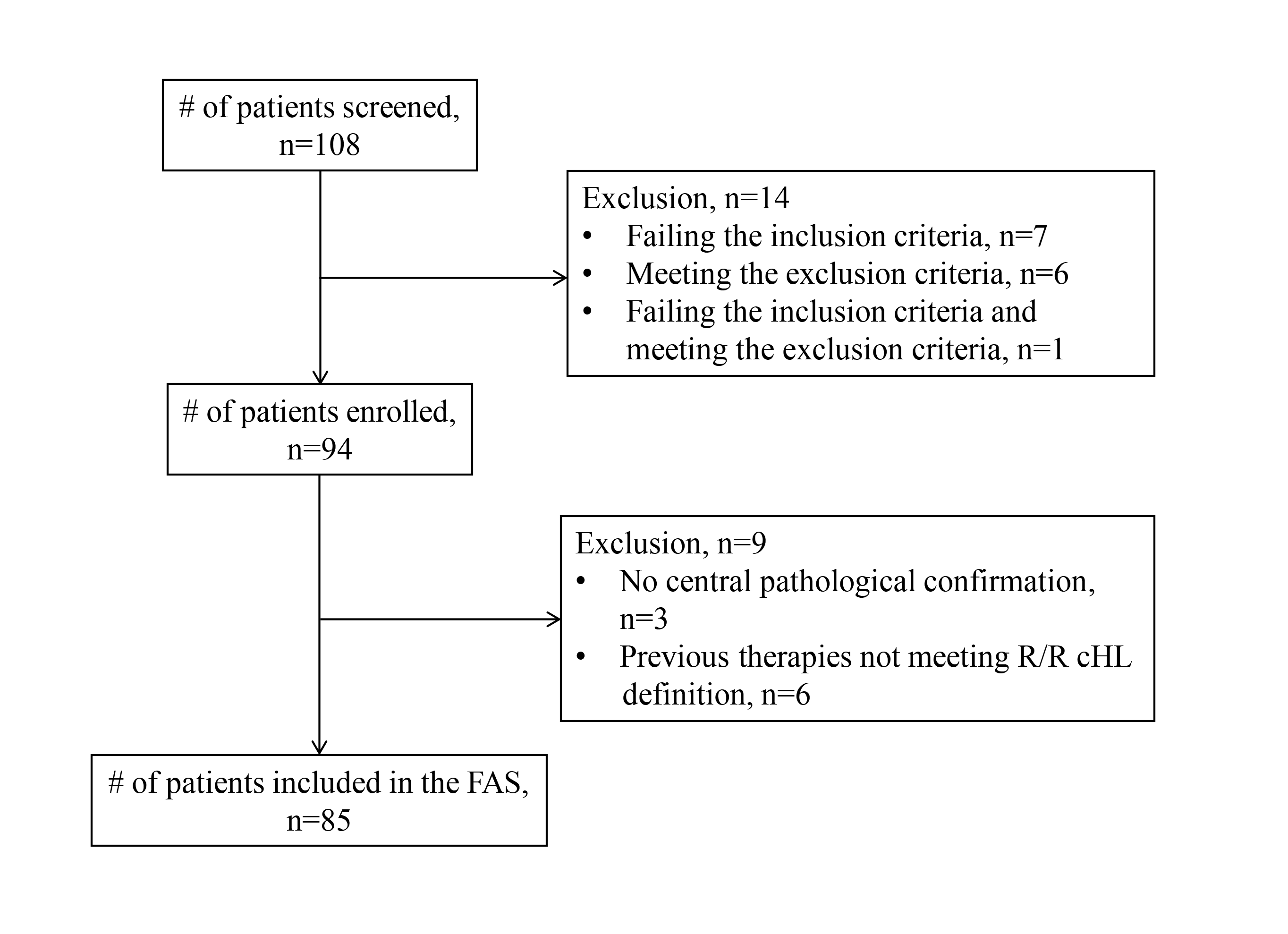

Supplement: Supplementary file 2 [file Image_1.tiff]

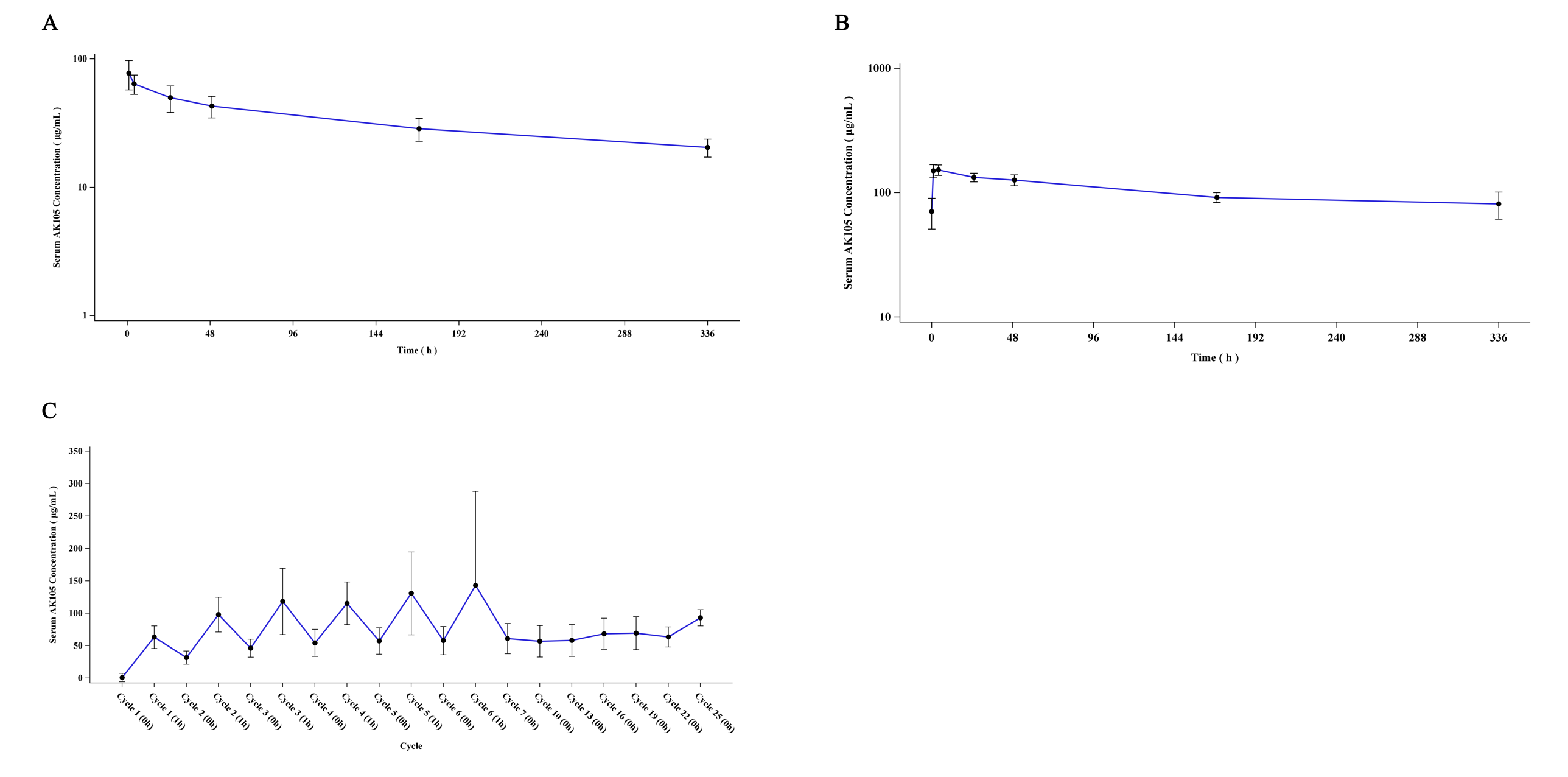

Supplement: Supplementary file 3 [file Image_2.tif]
