# Supplementary material for: Penpulimab for Relapsed or Refractory Classical Hodgkin Lymphoma: A Multicenter, Single-Arm, Pivotal Phase I/II Trial (AK105-201)
Source: Front Oncol. 2022 Jul 7;12:925236. doi: 10.3389/fonc.2022.925236 (PMC9301139; doi:10.3389/fonc.2022.925236)
Supplement: Supplementary file 4 [file Table_1.docx]

**Supplementary Table 1.** **Demographic and baseline characteristics of the study patients who had received 2 lines of prior chemotherapy**

|  |  |  |
| --- | --- | --- |
| Variables | | **N=40** |
| Age (y), median (range) | | 29.7(19,60) |
| Male sex | | 25 (62.5) |
| ECOG performance score | |  |
| 0 | | 33 (82.5) |
| 1 | | 7 (17.5) |
| B symptoms | | 19 (47.5) |
| Clinical stage | |  |
| I | | 1 (2.5) |
| II | | 7 (17.5) |
| III | | 7 (17.5) |
| IV | | 25 (62.5) |
| Histology | |  |
| Nodular sclerosing Hodgkin lymphoma (NSHL) | | 27 (67.5) |
| Mixed cellularity (MCHL) | | 9 (22.5) |
| Lymphocyte-rich classical HL (LRCHL) | | 3 (7.5) |
| Unknown | | 0 |
| Duration from initial diagnosis, median (range) | | 15.7 (3.4, 86.4) |
| Bone marrow infiltration | | 8 (20.0) |
| Previous chemotherapy | |  |
| Median (range) lines | | 2 (2,2) |
| ≥3 lines | | 0 |
| Other previous therapies | |  |
| Surgery | | 1 (2.5) |
| Radiotherapy | | 19 (47.5) |
| Autologous hematopoietic stem cell transplant | | 0 |
| Brentuximab vedotin | | 0 |
|  |  |  |
