# Supplementary material for: Penpulimab for Relapsed or Refractory Classical Hodgkin Lymphoma: A Multicenter, Single-Arm, Pivotal Phase I/II Trial (AK105-201)
Source: Front Oncol. 2022 Jul 7;12:925236. doi: 10.3389/fonc.2022.925236 (PMC9301139; doi:10.3389/fonc.2022.925236)
Supplement: Supplementary file 5 [file Table_2.docx]

**Supplementary Table 2 Drug exposure in the study population-the safety set (n=94)**

| Variables | Penpulimab (N=94) |
| --- | --- |
| Total exposure time, days | |
| Mean (SD) | 427.0 (146.1) |
| Median (Q1, Q3) | 449.0 (369.0, 505.0) |
| No. of infusions | |
| Mean (SD) | 27.4 (9.8) |
| Median (Q1, Q3) | 28.0 (21.0, 33.0) |
| Relative dose intensity, % | |
| Mean (SD) | 90.06 (9.09) |
| Median (Q1, Q3) | 91.36 (86.98,96.23) |
| Total exposure time, months | |
| ≥3 | 91 (96.8%) |
| ≥6 | 86 (91.5%) |
| ≥12 | 72 (76.6%) |
