# Supplementary material for: Penpulimab for Relapsed or Refractory Classical Hodgkin Lymphoma: A Multicenter, Single-Arm, Pivotal Phase I/II Trial (AK105-201)
Source: Front Oncol. 2022 Jul 7;12:925236. doi: 10.3389/fonc.2022.925236 (PMC9301139; doi:10.3389/fonc.2022.925236)
Supplement: Supplementary file 6 [file Table_3.docx]

**Supplementary Table 3 All grades, CTCAE grade 3 and above and severe immune-related adverse events (irAEs) and irAEs causing treatment interruptions or discontinuation in the safety set (n=94)**

| irAEs | irAEs, any grade, | irAEs, grade 3 and above | Severe irAEs | irAEs causing treatment interruptions | irAEs causing treatment discontinuation |
| --- | --- | --- | --- | --- | --- |
| Hypothyroidism | 27 (28.7) | - |  | 2 (2.1) |  |
| Elevated TSH | 10 (10.6) |  |  |  |  |
| Hyperthyroidism | 6 (6.4) |  |  |  |  |
| Elevated free T3 | 5 (5.3) |  |  |  |  |
| Rash | 4 (4.3) | 1 (1.1) |  | - |  |
| Reduced TSH | 3 (3.2) |  |  |  |  |
| Reduced free thyroxine | 3 (3.2) |  |  |  |  |
| Immune mediated pneumonitis | 2 (2.1) | 1 (1.1) | 1 (1.1) |  | 2 (2.1) |
| Decreased thyroxine | 2 (2.1) |  |  |  |  |
| Decreased T3 | 2 (2.1) |  |  |  |  |
| Elevated T3 | 1 (1.1) |  |  |  |  |
| Elevated free thyroxine | 1 (1.1) |  |  |  |  |
| Elevated thyroid hormones | 1 (1.1) |  |  |  |  |
| Abnormal thyroid function | 1 (1.1) |  |  |  |  |
| Autoimmune thyroiditis | 1 (1.1) |  |  | 1 (1.1) |  |
| Thyroid gland mass | 1 (1.1) |  |  | 1 (1.1) |  |
| Generalized rash | 1 (1.1) |  |  | 1 (1.1) |  |
| Psoriasis | 1 (1.1) | 1 (1.1) |  | 1 (1.1) |  |
| Mesangial proliferative glomerulonephritis | 1 (1.1) |  | 1 (1.1) | 1 (1.1) | 1 (1.1) |
| Nerve injury | 1 (1.1) |  |  |  |  |
| Elevated blood glucose | 1 (1.1) |  |  |  |  |
| Hyperglycemia | 1 (1.1) |  |  |  |  |
| Reduced ACTH | 1 (1.1) |  |  |  |  |
| Dermatitis | 1 (1.1) |  |  |  |  |
| Lichen planus | 1 (1.1) |  |  | 1 (1.1) |  |
| Interstitial pneumonia | 1 (1.1) |  |  |  |  |
| Kidney injury | 1 (1.1) | 1 (1.1) | 1 (1.1) |  | 1 (1.1) |

Data are expressed as n (%)
